# Sleep in Genetically Confirmed Pantothenate Kinase-Associated Neurodegeneration: A Video-Polysomnographic Study

**DOI:** 10.4061/2010/342834

**Published:** 2010-06-29

**Authors:** Maria Livia Fantini, Giovanni Cossu, Andrea Molari, Monia Cabinio, Ozlem Uyanik, Roberto Cilia, Maurizio Melis, Angelo Antonini, Luigi Ferini-Strambi

**Affiliations:** ^1^Sleep Disorders Center, Department of Clinical Neurosciences, Vita-Salute San Raffaele University, H San Raffaele-Turro, 20127 Milan, Italy; ^2^Department of Neuroscience, A.O.B. S. Michele Hospital, Cagliari 09121, Italy; ^3^Parkinson Institute, Istituti clinici di perfezionamento, Milan 20126, Italy; ^4^IRCCS, San Camillo, 30100 Venice, Italy

## Abstract

Pantothenate kinase-associated neurodegeneration (PKAN) is a familial or sporadic disease characterized by extrapyramidal and corticospinal signs with dementia. Patients show iron accumulation in the basal ganglia, with neuronal loss and gliosis. A mutation of pantothenate kinase (PANK2) gene localized on chromosome 20p13 has been described in familiar forms, as well as in sporadic patients. We sought to assess sleep characteristics, including muscle activity during REM sleep, in three patients with PANK2 gene mutation-confirmed diagnosis of PKAN. Sleep architecture was altered in all patients with reduced total time of sleep in two and lack of SWS in one. No significant apnea/hypopnea were detected, and mild PLMS were observed in one patient (PLMS index:10.7/h). In contrast with other neurodegenerative diseases, no REM sleep abnormalities, especially REM sleep behavior disorder, were observed in PKAN patients, and percentage of both REM sleep atonia and phasic EMG activity were within normal ranges. Sleep studies may phenotypically differentiate PKAN from other neurodegenerative disorders.

## 1. Introduction

Pantothenate kinase-associated neurodegeneration (PKAN), previously known as Hallervorden-Spatz disease, is a rare, progressive, familial or sporadic extrapyramidal dysfunction with dementia associated with iron accumulation in the basal ganglia. PKAN is an inherited condition recessively linked to chromosome 20 [1], and mutations in the pantothenate kinase 2 (PANK2) gene on chromosome 20p13 have been recently described in this disorder [2, 3].

PKAN patients show excessive iron deposition in the Globus Pallidus (GP) and Substantia Nigra pars reticulate (SNr) associated to neuronal loss and gliosis. Generalized brain atrophy including caudate nuclei, SN, and mesencephalic tegmentum may also occur. Tau-positive neurofibrillary tangles and alpha-synuclein-positive Lewy bodies may be found in cortical and subcortical regions in patients with prolonged clinical course [4].

PKAN is characterized by progressive extrapyramidal signs (rigidity, dystonia, and choreoathetosis), corticospinal signs (spasticity, hyperreflexia), and cognitive dysfunctions. Typical presentation symptoms include extrapyramidal signs, gait difficulty, and intellectual subnormalities, that most commonly occur in late childhood or early adolescence. Optic atrophy, retinitis pigmentosa, and seizures are accessory symptoms [3].

Changes in both macro-and microstructure of sleep and sleep disorders, such as REM sleep behavior disorder (RBD), Periodic Leg Movements (PLM), Restless Legs Syndrome (RLS), and Obstructive Sleep Apnea Syndrome (OSAS), have been associated with most extrapyramidal diseases including Parkinson's disease [5], Multiple System Atrophy [6], Cortico-Basal Degeneration [7, 8], Progressive Supranuclear Palsy [9], Lewy body dementia [10], Gilles de la Tourette syndrome [11], and Huntington's chorea [12].

Brainstem aminergic systems, including noradrenergic and serotoninergic projections, as well as mesocorticolimbic and nigrostriatal dopaminergic pathways, are involved in the sleep-wake cycle regulation and in behavioral state modulation, and basal ganglia are thought to be implicated in the complex mechanism regulating muscle atonia during REM sleep [13]. To our knowledge, sleep characteristics of patients with PKAN have not been reported so far. We sought to evaluate the sleep features, including the occurrence of abnormal muscle activity during sleep (REM sleep without atonia, RBD, and PLMS), in three patients diagnosed as PKAN based on the presence of a homozygous PANK2 gene mutation. 

## 2. Subjects and Methods

Patients 1 and 2 are two previously reported siblings of Sardinian origin diagnosed with PKAN. Patients 1 is a 25-year-old woman who developed at age 16 slurred speech, postural tremor in both hands (7-8 Hz), and increasing gait problems. She then showed dystonic posturing of both hands, mild action face dystonia and leg rigidity with balance impairment. Later her speech became dysarthric, a nasal voice appeared and a Babinski sign was revealed at neurological examination. Patient 2 developed hypophonia, slurred speech, and action dystonia in lower extremity at age 20. Later emergence of behavioral disturbance and impulse dyscontrol required antipsychotic medication (quetiapine 100 mg/day) discontinued after few months. In both patients, an extended neuropsychological evaluation revealed a frontosubcortical dysfunction. T2-weighted MRI showed decreased signal intensity in the pallidal nuclei with a centrally located area of increased intensity, giving the “eye-of-the tiger” appearance. Their SPECT with [123 I]FP-CIT revealed normal dopaminergic presynaptic function and showed central pallidum and SNr involvement [14]. These patients were homozygous for a missense mutation (556 C to T) in PANK2 gene, which results in an amino acid substitution (arginine to cysteine at position 176) [15]. The mutation found in these patients also occurred in one unrelated Italian patient who was a compound heterozygote [2]. Patient 3 clinical, genetic and imaging data have been previously published in [16]. He is a 54-year-old man who developed at age of 30 years personality changes associated with choreoathetosis and postural tremor of the right hand and leg. Because of behavioral problems he had been initially treated with neuroleptics (haloperidol and later pimozide) with partial benefit on psychiatric symptoms but with worsening of gait and postural instability associated with occasional falls. He then came to the neurological attention with bilateral bradykinesia, dystonia of upper and lower right limbs, mild postural tremor, freezing gait with severe postural instability and retropulsion, and severe dysarthria. Because of speech problems, his cognition could not be extensively investigated, but recognition and spatial-temporal orientation appeared normal. He also showed stereotyped movements, hyperreflexia and orofacial dyskinesias. On MRI T2-weighted images, pallidal nuclei showed the typical “eye-of-the tiger” feature. Additionally, SPECT imaging with [123 I]-Ioflupane (DaTSCAN), a dopamine transporter (DAT) tracer, showed a mild uniform DAT decrement on the right striatum with normal uptake values in the left striatum. Genetic studies revealed two heterozygous mutations in the PANK2 gene: 775 G > A and 1141 C > T. The first one was a missense mutation which resulted in an amino acid substitution (glycine replaced by arginine in position 259). The 1141 C > T was a nonsense mutation that leads to a truncated protein.

Patients 1 and 2 had no history of insomnia and their sleep schedule was regular at the time of the study. Patient 3 complained of occasional difficulties of initiating or maintaining sleep. None of the patients complained of RLS and there was no history of repetitive limb movements during sleep, violent motor behaviors associated to dream mentation or other parasomnia. Patients 1 and 2 were not taking psychotropic drugs at the time of the study, while patient 3 was taking levodopa (150 mg/day) and clonazepam (2 mg at noon and at bedtime) since at least two years, the latter because of the presence of myoclonic jerks. 

The study was approved by the local ethics committees and patients gave written informed consent. All patients underwent one night of Video-Polysomnographic (VPSG) recording in the sleep laboratory, after one adaptation night. Sleep was recorded and scored according to Rechtschaffen and Kales' method, using 30-second epochs. PSG included electroencephalographic recording (C3/A2, C4/A1, according to the 10–20 international electrode placement system), right and left electrooculogram, and chin electromyogram. Recording of oral and nasal air flow, thoracic and abdominal movements, and oximetry was performed to detect apneas or hypopneas. Surface EMG of the right and left anterior tibialis muscles was recorded to quantify leg movements. An additional surface EMG of right deltoid muscle was recorded to quantify upper limb movements. 

Electrocardiogram was recorded from a standard D2 lead. Periodic leg movements during sleep (PLMS) was recorded and scored according to standard criteria, that is, leg movements lasting 0,5 to 5 seconds, separated by intervals of 4 to 90 seconds and occurring in series of at least four consecutive movements. Microarousals were scored an the C3-A2 EEG lead using the ASDA criteria. Microarousal index (number per hour of sleep) was calculated, as well as the percentage of PLMS associated with microarousals (PLMS-MA). A PLMS was considered associated with microarousal when the latter started within 2 seconds before or after the onset of PLMS. Percentages of REM sleep atonia and of phasic chin EMG activity during REM sleep were measured, according to the method described by Lapierre and Montplaisir [17]. Furthermore, to better assess motor activity during REM sleep, phasic EMG activity of the upper and lower limbs was also recorded in the right deltoid muscle and in both tibialis muscles. Lower limb phasic activity was obtained by averaging the values of the activity recorded in both tibialis muscles.

## 3. Results

PSG characteristics are shown in Table 1. Total sleep time was reduced in both patients 1 and 2, with low sleep efficiency due to prolonged periods of wakefulness after sleep onset. The same variables were normal in patient 3. Percentage of SWS was normal to elevated in patients 1 and 2, while SWS was virtually missing in patient 3. Sleep spindle and K complex were normally represented in all patients, and microarousal index was within the normal range. None of the three patients was found to have apnea (AHI < 5) and oxyemoglobin saturation was always higher than 90% in all patients through the night. PLMS were not observed in patients 1 and 2. Patient 3 showed mild PLMS (PLMS index: 10.5/h) almost exclusively during NREM sleep, and rarely associated to microarousals. Beside PLMS, no other motor behaviors were observed during sleep in these patients. Tonic and phasic features of REM sleep are shown in Table 2. The totality of epochs of REM sleep were classified as atonic (mean % of REM sleep atonia: 100%) in all patients. There was a very low percentage of phasic EMG activity during REM sleep in either chin, upper and lower limbs muscles in all patient, with the mean values of 0.47, 0.43 and 2.70, respectively. Furthermore, no abnormal phasic EMG activity was observed during NREM sleep.

## 4. Discussion

Sleep architecture was altered in all PKAN patients. Indeed, the two unmedicated sibling patients showed a severe reduction of total time of sleep, with a normal and even increased percentage of SWS. The third patient showed a normal amount of sleep, with increased percentage of light sleep and no SWS. The latter finding may be partly ascribed to pharmacological effects, since benzodiazepines are known to reduce SWS, although these effects tend to decrease with chronic intake at stable doses, as was the case of patient 3. Alternatively, the lack of SWS in this patient could be related to older age, longer disease duration and severity of neurodegeneration. Indeed, differences in sleep pattern might reflect the clinical heterogeneity of the disease, since early and late onset PKAN differ in terms of clinical presentation. Despite alterations in macrostructure, no fragmentation of sleep microstructure, for example, increased number of microarousal, was observed. Nevertheless, in all patients no other sleep disorders were detected, particularly there were no significant apnea or hypopnea, and very mild PLMS were observed only in the oldest patient. Moreover, none of the subjects showed clinical episodes of REM sleep behavior disorder. Of interest, in contrast with other neurodegenerative diseases (particularly with most alpha-synucleinopathies), REM sleep atonia was found to be totally preserved, with a percentage of REM sleep atonic epochs of 100% in all patients. Phasic EMG activity during both REM and NREM sleep was also normal, and perhaps even decreased in these patients, when compared to published normal values [18]. REM sleep muscle atonia area, located mainly in the brainstem, are strictly interconnected with basal ganglia. Peduncolo-pontine nucleus (PPN), involved in the muscle tone inhibitory control during both wakefulness and sleep, receives inhibitory GABAergic projections from SNr and from the internal part of GP (GPi). Although no pathological data about these patients are available, it may be hypothesized that neuronal loss and iron inclusions usually observed in Gpi and in SNr in patients with PKAN would result in reduced inhibitory outputs to PPN with normal or even enhanced muscle atonia during REM sleep. 

This is the first description of sleep characteristics and REM sleep features in patients with PKAN. If replicated in a larger series of PKAN patients, the finding of a normal motor control during sleep might help in differentiating PKAN from other neurodegenerative conditions and may shed light on the role of basal ganglia in modulating REM sleep atonia.

## Figures and Tables

**Table 1:** Polysomnographic characteristics of patients with PKAN.

	Patient 1	Patient 2	Patient 3
Age	25	30	54
Gender	F	M	M
Age of onset of PKAN	16	20	30
Sleep latency (min.)	2.5	41	13
Total sleep time (min.)	250	280.5	384.9
N. of awakenings	9	10	3
Wake after sleep onset (WASO)	177	141	10.5
Sleep efficiency (%)	58.6	60.6	94.2
% Stage 1 sleep	3.1	8.2	1.2
% Stage 2 sleep	53.3	55.4	77.2
% SWS	26.4	27.7	0.3
% Stage REM sleep	17.2	8.7	21.4
N. of REM periods	4	2	2
MA index	4.6	7.3	10.1
PLMS index	0	0	10.9
% PLMS associated with MA	—	—	21.6
AHI	0	0	1.5

SWS: Slow Wave Sleep; REM: Rapid Eye Movements; MA: Microarousals; PLMS: Periodic Leg Movements during sleep; PLMW: Periodic Leg Movements during wakefulness; AHI: Apnea-Hypopnea Index.

**Table 2:** Polysomnographic features of REM sleep in PKAN.

	Patient 1	Patient 2	Patient 3
% REM sleep atonia	100	100	100
% phasic EMG activity			
chin	0.3	0	1.4
deltoids	0	0.1	1.2
tibialis	0.6	2.7	4.8
